# Predicting preeclampsia from a history of preterm birth

**DOI:** 10.1371/journal.pone.0181016

**Published:** 2017-07-24

**Authors:** Svein Rasmussen, Cathrine Ebbing, Lorentz M. Irgens

**Affiliations:** 1 Department of Clinical Science, University of Bergen, Bergen, Norway; 2 Department of Obstetrics and Gynaecology, Haukeland University Hospital, Bergen, Norway; 3 Department of Global Public Health and Primary Care, University of Bergen, Bergen, Norway; 4 Medical Birth Registry of Norway, Norwegian Institute of Public Health, Bergen, Norway; University of Missouri Columbia, UNITED STATES

## Abstract

**Objective:**

To assess whether women with a history of preterm birth, independent on the presence of prelabour rupture of the membranes (PROM) and growth deviation of the newborn, are more likely to develop preeclampsia with preterm or preterm birth in a subsequent pregnancy.

**Methods:**

We conducted a population-based cohort study, based on Medical Birth Registry of Norway between 1967 and 2012, including 742,980 women with singleton pregnancies who were followed up from their 1st to 2nd pregnancy. In the analyses we included 712,511 women after excluding 30,469 women with preeclampsia in the first pregnancy.

**Results:**

After preterm birth without preeclampsia in the first pregnancy, the risk of preterm preeclampsia in the second pregnancy was 4–7 fold higher than after term birth (odds ratios 3.5; 95% confidence interval (CI) 3.0–4.0 to 6.5; 95% CI 5.1–8.2). The risk of term preeclampsia in the pregnancy following a preterm birth was 2–3 times higher than after term birth (odds ratios 1.6; 95% CI 1.5–1.8 to 2.6; 95% CI 2.0–3.4). After spontaneous non-PROM preterm birth and preterm PROM, the risk of preterm preeclampsia was 3.3–3.6 fold higher than after spontaneous term birth. Corresponding risks of term preeclampsia was 1.6–1.8 fold higher. No significant time trends were found in the effect of spontaneous preterm birth in the first pregnancy on preterm or term preeclampsia in the second pregnancy.

**Conclusions:**

The results suggest that preterm birth, regardless of the presence of PROM, and preeclampsia share pathophysiologic mechanisms. These mechanisms may cause preterm birth in one pregnancy and preeclampsia in a subsequent pregnancy in the same woman. The association was particularly evident with preterm preeclampsia.

## Introduction

Growing evidence suggests that some cases of preterm birth, preterm prelabour rupture of the membranes (PROM), foetal growth restriction, preeclampsia and placental abruption share a pathophysiological mechanism involving placental dysfunction [1–4].

Another cause of preterm birth is inflammation of the placenta or membranes (chronic or acute chorioamnionitis [1, 2], chronic nonspecific villitis and chronic deciduitis) [5]. New evidence indicates that a generalized inflammatory response in pregnant women may predispose to preterm birth with or without PROM [5–7] and preeclampsia [3, 8].

Furthermore, preterm birth and preeclampsia tend to recur [9–11]. Thus, one may hypothesize that recurrent placental dysfunction and chorioamnionitis may predispose to preterm birth, with or without PROM, in the first pregnancy followed by a pregnancy complicated by preeclampsia in the next. We have earlier reported that having a small for gestational age infant in the first pregnancy is associated with preeclampsia [12–14], particularly severe preeclampsia [13], in a subsequent pregnancy.

An excess risk of preeclampsia subsequent to a preterm birth has been reported [15]. However, it is not known if preterm birth is more predictive of preterm than term preeclampsia in the subsequent pregnancy. Neither has the possible effect of preterm birth with or without PROM on later preeclampsia been studied.

In the present study, we assessed whether women with a history of preterm birth, regardless of growth deviation of the newborn, are more likely to develop preeclampsia with preterm or term birth. A second aim was to investigate whether the presence of preterm PROM in the first pregnancy influences the risk of later preeclampsia.

## Materials and methods

From 1967 to 2012 a total of 2,707,724 births were recorded in the national Medical Birth Registry of Norway. Using the mothers’ national identification number, the first and second births were linked. In the present study we included women with at least two singleton births with gestational age between 16 and 44 weeks (*n* = 742,980). In the analyses we included 712,511 women, excluding 30,469 women with preeclampsia in the first pregnancy.

The outcome variable in the second pregnancy was preeclampsia, further categorized into term (with birth ≥37 weeks of gestation) and preterm (<37 weeks) preeclampsia. In Norway, the diagnosis of preeclampsia is in accordance with the recommendations of the American College of Obstetricians and Gynecologists [16] which defined preeclampsia as the presence of systolic blood pressure of ≥140 mmHg or a diastolic pressure of ≥90 mmHg on two occasions at least 4 hours apart after 20 weeks of gestation in a woman with a previously normal blood pressure with proteinuria defined as ≥0.3 g/ hour urine collection, protein/creatinine ratio ≥0.3, or dipstick reading 1+.

Possible predictors in the first pregnancy were gestational age at birth (16–21, 22–27, 28–32, 33–36, 37–44 weeks) and preterm PROM. This extensive gestational age range allowed study of effects of second trimester miscarriage as well as extreme preterm delivery (<28 weeks), very preterm delivery (28–32 weeks) and near term preterm delivery (33–36) on later preeclampsia. Gestational age was estimated from the last menstrual period. From December 1998, gestational age based on ultrasound dating was available and was used when data on the last menstrual period were lacking. Until 1998 PROM was notified by plain text as rupture of the membranes more than 24 hours before birth, and from 1998 by a check box. Premature rupture of membranes occurring before 37 weeks of gestation was referred to as preterm PROM.

The associations between gestational age in the first pregnancy and preeclampsia in the second were assessed by odds ratios (ORs) obtained by forward stepwise logistic regression analyses. The following possible confounders in the second pregnancy were entered in the regression models: Birthweight <10th and >90th centiles (based on stratification for gestational age in weeks of gestation from 22 to 44 weeks, birth order; 1 and 2+, and foetal gender), maternal age (<20, 5-year increments, 40 years+), marital status (married or cohabiting, other), inter-birth interval (<5, 5–9, 10 years+), asthma, chronic hypertension, chronic renal disease, rheumatoid arthritis, maternal cardiac disease, gestational diabetes- or pre-gestational diabetes and year of birth (1967–75, 1976–84, 1985–93, 1994–2002, 2003–12).

From 1998 smoking was recorded. We included smoking at the beginning of the second pregnancy in the analyses, categorized as no, occasionally, daily and not specified. From 2007 data on maternal body mass index (BMI) were registered in 38% of the second births (*n* = 109, 958). Maternal pre pregnant BMI was categorized according to the definitions of the World Health Organization into <18.5 kg/m^2^ (underweight), 18.5–24.9 (normal), 25.0–29.9 (overweight) and ≥30.0 (obese).

To assess if the association between a history of spontaneous preterm birth and the occurrence of preeclampsia differed with time period, we added year of the first birth in an interaction term in the regression model between spontaneous preterm birth in the first pregnancy and year of first births categorized into1967–80, 1981–94 and 1995–2008, following each cohort up to 18 years.

The statistical analysis was carried out with SPSS version 23 (Statistical Package for the Social Sciences; SPSS Inc, Chicago, IL. USA).

The study was approved by the Regional Ethics Committee for Medical and Health Research Ethics (REK nord case no. 2013/1200).

## Results

In the first and second pregnancies, the occurrence of preeclampsia was 30,469/742, 980 (4.1%) and 14,645/742,980 (2.0%), respectively. Women with preeclampsia in the second pregnancy tended to be older, had higher BMI and had more often chronic disease and gestational diabetes mellitus (Table 1). This pattern was generally more marked for preterm than term preeclampsia. Also, fewer smokers were observed among women with preeclampsia. There was no significant difference in marital status between women with and without preeclampsia.

**Table 1:** Background characteristics of women who had two successive singleton infants without preeclampsia in the first pregnancy and numbers and proportions in the second pregnancy of preterm preeclampsia (<37 weeks of gestation), term preeclampsia and no preeclampsia, Norway1967 to 2012. (*n* = 712511).

Characteristics in			Second pregnancy			
second pregnancy	Preeclampsia				No preeclampsia	
	<37 weeks (*n* = 1614)		37+ weeks (*n* = 8409)		(*n* = 702488)	
	*n*	%	*n*	%	*n*	%
**Maternal age (years)**						
<20	7	0.4	49	0.6	7983	1.1
20–24	186	11.5	1370	16.3	154945	22.1
25–29	551	34.1	3155	37.5	281413	40.1
30–34	546	33.8	2689	32.0	193340	27.5
35–39	274	17.0	978	11.6	57323	8.2
40+	50	3.1	168	2.0	7484	1.1
**Marital status**						
married/cohabiting	1518	94.1	7973	94.8	666296	94.8
other	96	5.9	436	5.2	36192	5.2
Smoking*						
no	571	70.2	2469	74.1	173636	71.3
occasionally	8	1.0	48	1.4	3680	1.5
daily	86	10.6	319	9.6	27169	11.2
not specified	148	18.2	496	14.9	38920	16.0
**Chronic maternal disease**						
Asthma	62	3.8	278	3.3	16192	2.3
Chronic hypertension	54	3.3	190	2.3	1548	0.2
Chronic renal disease	23	1.4	112	1.3	5839	0.8
Rheumatoid arthritis	10	0.6	29	0.3	1997	0.3
Maternal hearth disease	10	0.6	39	0.5	1962	0.3
Epilepsy	19	1.2	59	0.7	3910	0.6
Thyroid disease	28	1.7	102	1.2	6276	0.9
**Body mass index before pregnancy (kg/m**^**2**^)**						
<18.5	3	2.4	7	1.3	1525	3.7
18.5–24.9	47	37.9	210	38.3	25472	61.5
25–29.9	36	29.0	162	29.6	9588	23.1
30+	38	30.6	169	30.8	4843	11.7
**Diabetes, all types**	84	5.2	240	2.9	5862	0.8
Diabetes mellitus type 1*	24	3.0	43	1.3	939	0.4
Diabetes mellitus type 2*	8	1.0	24	0.7	435	0.2
Gestational diabetes*	18	2.2	96	2.9	2477	1.0
**Gestational age (weeks), 1st pregnancy**						
16–21	18	1.1	57	0.7	1874	0.3
22–27	37	2.3	67	0.8	3128	0.4
28–32	77	4.8	157	1.9	6041	0.9
33–36	194	12.0	537	6.4	28579	4.1
37–44	1288	79.8	7591	90.3	662866	94.4
**Birthweight centile, 1st pregnancy**						
<10	320	19.8	984	11.7	65104	9.3
10–90	1175	72.8	6561	78.0	567427	80.8
>90	109	6.8	836	9.9	69127	9.8
unclassified	10	0.6	28	0.3	830	0.1

*Dec. 1998 to 2012

**2007 to 2012.

**Fig 1:**
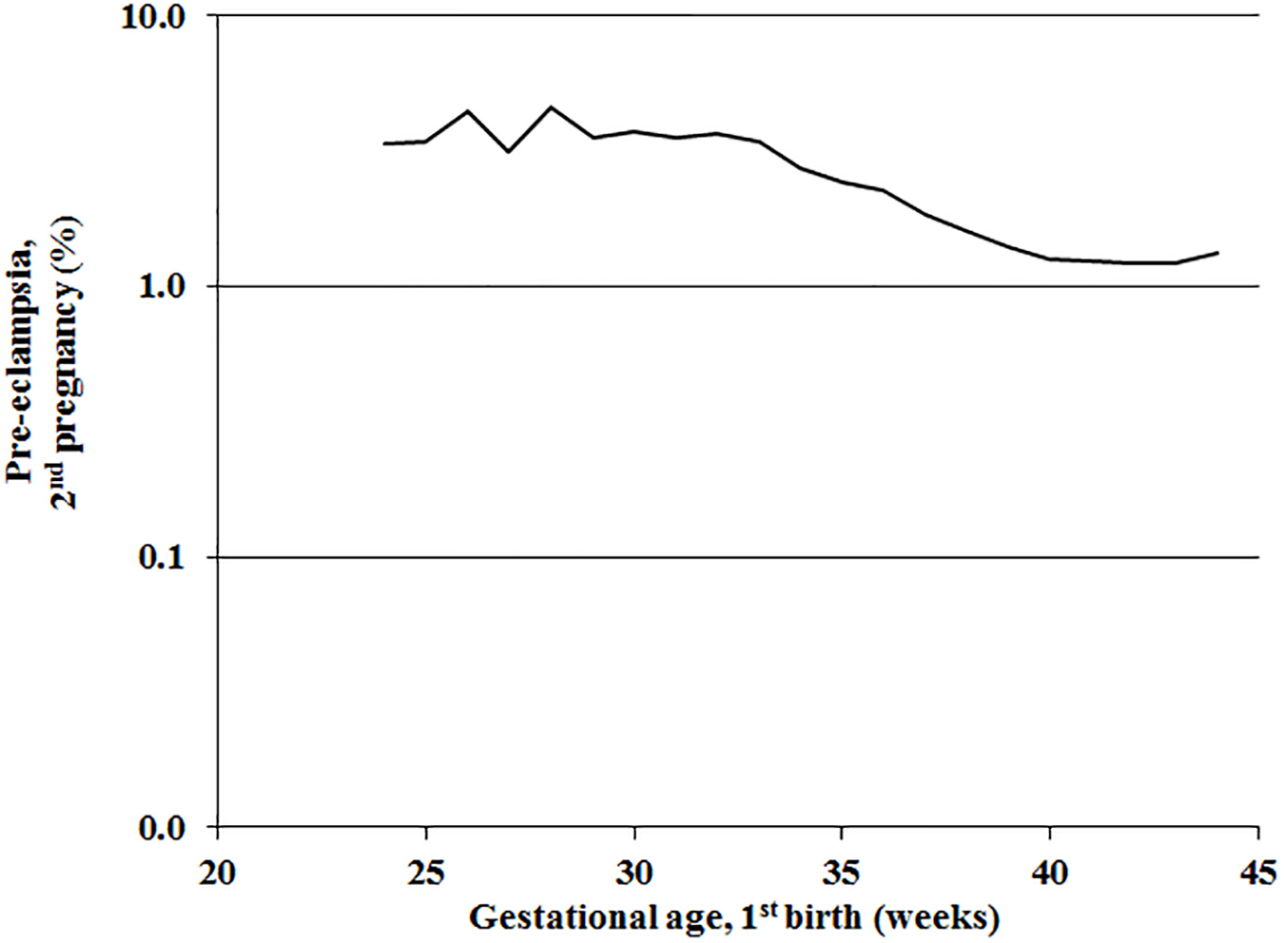
Preelampsia in the second pregnancy according to gestational age at the first birth in the same woman without preeclampsia in the first pregnancy. Norway 1967–2012.

In women without preeclampsia in the first pregnancy the occurrence of preeclampsia in the second pregnancy generally increased with decreasing gestational age at birth in the first pregnancy (Fig 1).

After preterm birth in the first pregnancy, preterm and term preeclampsia were 4–7 and 2–3 times more likely to occur in the second pregnancy compared with after term birth, respectively (Table 2). These associations persisted after adjusting for confounders, including birth weight percentile in the first birth (Table 2) and in stratified analyses of births at or below and above the 10^th^ birth weight centile.

**Table 2:** Preterm and term preeclampsia in the second pregnancy according to gestational age in the first birth, 1998–2012. (*n* = 712511).

1st pregnancy		Preterm preeclampsia in 2nd pregnancy						Term preeclampsia in 2nd pregnancy					
Gestational	Total	*n*	%	OR	95% CI	OR*	95% CI	*n*	%	OR	95% CI	OR**	95% CI
age (weeks)													
16–21	1949	18	0.9	4.9	(3.0–7.7)	4.1	(2.6–6.6)	57	2.9	2.6	(2.0–3.4)	2.7	(2.0–3.5)
22–27	3232	37	1.1	6.0	(4.3–8.4)	5.5	(4.0–7.8)	67	2.1	1.9	(1.5–2.4)	1.8	(1.4–2.3)
28–32	6275	77	1.2	6.5	(5.1–8.2)	5.6	(4.4–7.0)	157	2.5	2.2	(1.9–2.6)	2.1	(1.8–2.5)
33–36	29310	194	0.7	3.5	(3.0–4.0)	3.1	(2.7–3.6)	537	1.8	1.6	(1.5–1.8)	1.6	(1.4–1.7)
37–44	671745	1288	0.2	Reference		Reference		7591	1.1	Reference		Reference	

*Adjusted for the following variables in the 2nd pregnancy: maternal age, chronic hypertension, epilepsy, gestational- and pre-pregnancy diabetes

birthweight centile <10 and >90, year of delivery and inter-birth interval.

**Adjusted for the following variables in the 2nd pregnancy: maternal age, chronic hypertension, chronic renal disease, gestational- and pre-pregnancy

diabetes and birthweight centile <10 and >90.

OR = odds ratio; CI = confidence interval.

Restricting the analysis to spontaneous births in the first pregnancy, the associations persisted, but were less pronounced (Table 3); after preterm birth in the first pregnancy, preterm and term preeclampsia were 3–4 and about 2 times more likely to occur in the second pregnancy than after term births, respectively.

**Table 3:** Preterm and term preeclampsia in the second pregnancy according to gestational age in the first birth, 1998–2012. 609962 women with spontaneous first deliveries. (*n* = 609962).

1st pregnancy		Preterm preeclampsia in 2nd pregnancy						Term preeclampsia in 2nd pregnancy					
Gestational age (weeks)	Total	*n*	%	OR	95% CI	OR*	95% CI	*n*	%	OR	95% CI		OR** 95% CI
16–21	791	4	0.5	2.9	(1.1–7.8)	2.8	(1.0–7.6)	18	2.9	2.2	(2.0–3.4)	2.7	(2.0–3.5)
22–27	2483	17	0.7	4.0	(2.4–6.4)	3.9	(2.4–6.4)	50	2.1	2.0	(1.5–2.4)	1.8	(1.4–2.3)
28–32	4938	37	0.7	4.3	(3.1–6.0)	3.9	(2.8–5.5)	92	2.5	1.8	(1.9–2.6)	2.1	(1.8–2.5)
33–36	24635	120	0.5	2.8	(2.3–3.4)	2.6	(2.1–3.1)	377	1.8	1.5	(1.5–1.8)	1.6	(1.4–1.7)
37–44	577115	1005	0.2	Reference		Reference		5966	1.0	Reference		Reference	

*Adjusted for the following variables in the 2nd pregnancy: maternal age, chronic hypertension, gestational- and pre-pregnancy diabetes

birthweight centile <10 and >90, year of delivery and inter-birth interval.

**Adjusted for the following variables in the 2nd pregnancy: maternal age, chronic hypertension, chronic renal disease, asthma, gestational- and

pre-pregnancy diabetes, birthweight centile <10 and >90, year of delivery and inter-birth interval.

OR = odds ratio; CI = confidence interval.

Restricting the analysis to 153,009 women with spontaneous first births after 1998 when the new notification form was introduced, and PROM was more completely recorded as indicated by higher frequencies of PROM, we assessed the possible associations of spontaneous preterm birth without and with PROM in the first pregnancy with preterm and term preeclampsia in the second. The effects of spontaneous preterm birth with and without PROM were similar (Table 4). After spontaneous non-PROM preterm birth and preterm PROM, the risk of preterm preeclampsia was 3.3–3.6 fold higher than after spontaneous term birth, while the risks of term preeclampsia was 1.6–1.8 fold higher. Adjusting for possible confounders did not change the results. Adjusting for maternal BMI (*n* = 42,089) had negligible effect; for preterm and term delivery in the first pregnancy proportions of preeclampsia in the second were 0.8% (19/2299) and 0.3% (105/39790), respectively, with unadjusted and adjusted ORs of 3.2; 95% CI 1.9–5.1 and 3.1; 1.9–5.1.

**Table 4:** Preterm and term preeclampsia in the second pregnancy according to gestational age and PROM in the first birth, 1998–2012. (*n* = 153009).

1st pregnancy		Preterm preeclampsia in 2nd pregnancy						Term preeclampsia in 2nd pregnancy					
	Total	*n*	%	OR	95% CI	OR*	95% CI	*n*	%	OR	95% CI	OR**	95% CI
Spontaneous term birth	145141	287	0.2	Reference		Reference		1394	1.0	Reference		Reference	
Spontaneous non-PROM preterm birth	6172	44	0.7	3.6	(2.6–5.0)	3.3	(2.4–4.5)	110	1.8	1.9	(1.5–2.3)	1.8	(1.5–2.2)
Preterm PROM	1696	11	0.6	3.3	(1.8–6.0)	2.8	(1.5–5.2)	27	1.6	1.7	(1.1–2.4)	1.6	(1.1–2.3)

*Adjusted for the following variables in the 2nd pregnancy: maternal age, chronic hypertension, chronic renal disease, diabetes mellitus, birthweight centile <10 and >90 and inter-birth interval.

**Adjusted for the following variables in the 2nd pregnancy: marital status, chronic hypertension, diabetes mellitus, birthweight centile <10 and >90 and inter-birth interval.

PROM = preterm prelabour rupture of the membranes; OR = odds ratio; CI = confidence interval.

No significant time trends were found in the effect of spontaneous preterm birth in the first pregnancy on preterm or term preeclampsia in the second pregnancy.

## Discussion

### Main findings

This population based study indicates that a history of preterm birth irrespective of PROM, and regardless growth deviation of the first born, is associated with preeclampsia and particularly preterm preeclampsia in a following pregnancy.

### Strengths and limitations

Strengths of the study include the prospective collection of data and its population-based design. Furthermore, our database holds data on several possible confounders. Preeclampsia is associated with a number of risk factors such as maternal age, previous preeclampsia, chronic hypertension, pre-gestational diabetes, asthma, renal disease, thyroid disease, rheumatoid arthritis and maternal obesity. It is negatively associated with smoking. However, adjusting for these variables and year of birth in the subsequent pregnancy had small effects. Another strength is high validity of the outcome [17] and other variables used in the analyses [18]. However, before oedema was eliminated as a diagnostic criterion for preeclampsia some cases lacking proteinuria might have been notified as preeclampsia. Still, a study [17] showed that the validity according to the set diagnostic criteria of preeclampsia in the MBRN was high and stable during 1967–2002. In the Norwegian Mother and Child Cohort Study and the Medical Birth Registry of Norway, distributions of preterm birth, preeclampsia and other variables used in our study were similar [18], indicating high validity of the data.

That data on maternal BMI were available only in a subset of women represented a limitation. However, we have shown that this subset is representative of the whole Norwegian birth population [19]. Data on smoking were only available from 1998. However, adjusting smoking had no effect. Another limitation is the lack of data on ethnicity. Immigrant women have more often preterm birth [20], but less often preeclampsia [21]. Thus, the association between preterm birth and later preeclampsia would likely not be caused by failure of adjusting for ethnicity. Neither would the association be caused by low socioeconomic level, being only weakly or not associated with preeclampsia [22]. Because of lack of data, we were not able to address the possible paternal influence on the association of preterm birth with subsequent preeclampsia. In previous studies, we found a significant paternal effect on recurrence of preeclampsia [9], but not on preeclampsia in women with a history of delivery of a small for gestational age infant [13].

### Comparison with other studies

Our observation that preterm birth is associated with subsequent preeclampsia is consistent with another population based study [15]. A new finding in the present study was that preterm birth, regardless of PROM, is more predictive of preterm- than term preeclampsia. The finding that previous preterm birth is associated not only with preterm, but also with term preeclampsia, suggests that the association with preterm preeclampsia is not merely caused by the well-known high recurrence of preterm birth alone.

### Interpretation

The finding that spontaneous preterm birth with and without PROM was predictive of later preeclampsia, supports a hypothesis that a common pathophysiologic mechanism may cause preterm birth in one pregnancy and preterm as well as term preeclampsia in the next. A significant proportion of preterm births is caused by improper remodelling of the uterine spiral arteries in early pregnancy [2]. These decidual changes may lead to reduced placental perfusion, cause preterm birth as well as subsequent dysfunction of the maternal vascular endothelium. Thus, some cases of preterm birth may differ from preeclampsia only in the maternal response to a shared pathophysiological mechanism.

Another possible shared mechanism which may cause both preterm labour and preeclampsia is generalized systemic inflammation with chorioamnionitis (microbiological or non-microbiological), which also may be associated with endothelial dysfunction [7, 8]. Consistent with this are earlier findings of increased levels of systemic inflammatory markers in spontaneous preterm delivery as well as preeclampsia [3, 6, 8, 23] and excessive mortality from cardiovascular causes in women with a history of preterm birth or preeclampsia.[24]

Also local microorganisms in the placenta may cause preterm birth and preeclampsia. In preterm birth bacterial infection in the placenta, membranes and amniotic cavity have been observed [25]. Recent studies using culture independent techniques indicate that an apparently sterile placenta and possibly also implantation site are normally colonized by polymicrobial bacteria (microbiota) [25, 26]. In contrast to overt infection, such colonization may be beneficial to the normal development of pregnancy and may facilitate decidual trophoblast invasion. However, the presence of microorganisms alone is not sufficient to induce the inflammatory cascade leading to preterm parturition. Although little is known about the pathophysiological mechanisms involved, recent studies have supported the ‘double hit hypothesis’ that a viral infection of the placenta changes the placental response to local bacteria, increasing the risk of an inflammatory response resulting in preterm birth [26, 27].

## Conclusions

Our study suggests that preterm birth, regardless of the presence of PROM, and preeclampsia share pathophysiologic mechanisms. These mechanisms may cause preterm birth in one pregnancy and preeclampsia in a subsequent pregnancy in the same woman. The association was particularly evident with preterm preeclampsia. Future longitudinal studies should elucidate the relative importance of recurrent decidual vasculopathy and infection or inflammation in the development of preeclampsia in women with a history of preterm birth. Genetic or large-scale family studies are needed to assess possible paternal genetic influence on the association between preterm birth and subsequent preeclampsia, e.g. assessing whether men who had fathered a pregnancy with preterm birth are more likely to father a pregnancy with PE in another woman. Whether a history of preeclampsia involves an excess risk of preterm birth in a subsequent pregnancy also warrants study.
